# Hydrothermal Humification for Producing Humic-like Acids and Nitrogen-Enriched Humic-like Acids from Recycled Wheat Straw CTMP Black Liquor

**DOI:** 10.3390/polym18131629

**Published:** 2026-06-30

**Authors:** Xiaoyue Xu, Yichen Liu, Jiangtao Hu, Junlong Song, Wenyuan Zhu

**Affiliations:** 1 Jiangsu Provincial Key Lab of Sustainable Pulp and Paper Technology and Biomass Materials, Nanjing Forestry University, Nanjing 210037, China; xuxiaoyue@njfu.edu.cn (X.X.); yichenliu@njfu.edu.cn (Y.L.); junlong.song@njfu.edu.cn (J.S.); 2 Anhui Winbon New Materials Co., Ltd., Huangshan 245200, China; hjt@winbon-schoeller.com

**Keywords:** recycled wheat straw CTMP black liquor, hydrothermal humification, nitrogen-enriched humic-like acids, urea modification

## Abstract

The high-value utilization of non-wood pulping black liquor is of great significance for the sustainable development of the pulp and paper industry. In this study, concentrated black liquor, obtained from the five-time recycling of KOH-pretreated wheat straw chemi-thermomechanical pulp (CTMP), was used as the feedstock for the preparation of humic-like acids (HLAs) through hydrothermal humification by utilizing the enriched organic components and residual alkalinity. Urea was further introduced to synthesize nitrogen-enriched humic-like acids (N-HLAs). The hydrothermal conditions and urea dosage were systematically optimized, and the products were characterized by elemental analysis (EA), Fourier transform infrared spectroscopy (FT-IR), X-ray photoelectron spectroscopy (XPS), scanning electron microscopy (SEM), and particle size analysis (PSA). The results showed that the optimal hydrothermal condition was 180 °C for 4 h, under which the HLA yield reached 15.75%. With the addition of 1 mol/L urea, the yield of 1N-HLA further increased to 16.81%. Structural analyses demonstrated that hydrothermal treatment promoted the transformation of small molecular organics into highly aromatic and condensed macromolecular structures, while nitrogen-containing functional groups were successfully incorporated into the HLA molecular framework through urea modification. Bioactivity assay results showed that 1N-HLA exhibited a promoting effect on radish seed germination and seedling growth at a concentration of 100 mg/L. This study provides theoretical and technical support for the valorization of pulping black liquor and the green synthesis of functional humic-like materials.

## 1. Introduction

Humic acid (HA) is a naturally occurring macromolecular organic substance widely distributed in soils, sediments, and aquatic environments [1,2]. Owing to its abundant oxygen-containing functional groups, such as carboxyl, phenolic hydroxyl, and carbonyl groups [3], HA exhibits excellent ion-exchange, complexation, and redox properties, enabling broad applications in agriculture, environmental remediation, and functional material development [4,5,6].

Traditionally, humic acid is extracted from geological resources such as peat and lignite through alkali dissolution and acid precipitation processes. However, these feedstocks are non-renewable and require extremely long geological formation periods. Moreover, conventional extraction processes are often associated with considerable energy consumption and environmental burdens, which limit their sustainability and large-scale application [7,8]. To address these limitations, increasing attention has been directed toward the preparation of humic-like acids (HLAs) from renewable biomass resources and organic wastes [9]. Among the available technologies, hydrothermal humification (HTH) has emerged as a promising approach because it can rapidly convert biomass-derived organic matter into humic-like substances within several hours under relatively mild reaction conditions [10,11]. During hydrothermal treatment, lignocellulosic components are transformed into reactive intermediates that subsequently undergo condensation, dehydration, aromatization, and polymerization reactions, leading to the formation of humic-like macromolecular structures [12]. Compared with natural humification, hydrothermal humification significantly shortens the conversion period and improves carbon resource utilization efficiency. Lu et al. developed a two-step hydrothermal process, which could convert corn stalks into humic acid at 200 °C within 6 h, achieving a high humic acid yield of 67.9 wt% and excellent carbon retention efficiency [13]. Shao et al. demonstrated that oxygen-assisted hydrothermal humification of hydrochar at 180 °C for 4 h increased the humic acid yield from 41.9 wt% to 49.2 wt%, which effectively elevated carbon retention and reduced the loss of organic carbon [14]. Despite these advantages, several challenges remain in current HTH-based HLA production systems. Many reported processes require the addition of alkaline reagents to promote lignin depolymerization and humification reactions, which increases chemical consumption and operating costs. Furthermore, most biomass-derived HLAs contain limited nitrogen functionalities, restricting their biological activity and potential agricultural applications. Therefore, developing a cost-effective hydrothermal humification system capable of simultaneously utilizing inherent alkalinity and introducing nitrogen-containing functional groups remains an important research objective.

Nitrogen doping has been recognized as an effective strategy for enhancing the functionality of humic-like substances. Urea is an inexpensive and widely available nitrogen source that can generate reactive nitrogen-containing intermediates during hydrothermal treatment. These intermediates can participate in condensation reactions with carbonyl-containing compounds, promoting the incorporation of nitrogen-containing functional groups into humic-like structures [15,16]. Nitrogen-enriched humic-like acids (N-HLAs) have been reported to exhibit improved nutrient retention capacity, enhanced biological activity, and greater environmental responsiveness compared with conventional humic substances [17]. Pulping black liquor is a complex alkaline by-product rich in lignin-derived fragments, carbohydrates degradation products, and dissolved organic compounds [18]. Particularly in non-wood pulping systems, black liquor treatment remains a significant challenge because conventional alkali recovery processes are severely hindered by high silica content, resulting in poor recovery efficiency and limited economic benefits [19,20]. Nevertheless, the abundant organic matter and residual alkalinity present in black liquor make it a potentially attractive feedstock for hydrothermal humification. Unlike many previously reported biomass-based systems that require external alkaline additives, pulping black liquor can simultaneously serve as both a carbon source and an alkaline reaction medium, thereby reducing chemical inputs and simplifying the process. Although several studies have explored the preparation of humic-like substances from biomass residues and organic wastes, the conversion of non-wood pulping black liquor into nitrogen-enriched humic-like acids through hydrothermal humification has received limited attention. Furthermore, the effects of hydrothermal conditions and nitrogen doping on product formation, structural evolution, and biological activity remain insufficiently understood.

Therefore, this study employed concentrated wheat straw CTMP black liquor (pH = 9.5) as a feedstock for the preparation of humic-like acids through hydrothermal humification without the addition of external alkaline reagents. The effects of reaction temperature and residence time on product yield and structural characteristics were systematically investigated. Urea was further introduced as a nitrogen source to prepare nitrogen-enriched humic-like acids (N-HLAs), and advanced characterization techniques including elemental analysis (EA), Fourier transform infrared spectroscopy (FTIR), and X-ray photoelectron spectroscopy (XPS) were employed to elucidate the effects of nitrogen doping on the molecular structure and functional group distribution of the products. In addition, radish seed germination and seedling growth experiments were conducted to assess the biostimulatory activity of the obtained products. This work provides a sustainable and value-added utilization pathway for non-wood pulping black liquor while contributing to the development of functional humic-like materials from industrial biomass-derived residues.

## 2. Materials and Methods

### 2.1. Materials

Recycled and concentrated wheat straw CTMP black liquor: A total of 100 g of oven-dried wheat straw (Huisheng Paper Industry, Dezhou, China) was pretreated using 7% KOH with a liquid-to-solid ratio of 4:1 in a 15 L laboratory-scale vertical rotary digester at 100 °C for 45 min. After pretreatment, the pulp slurry was separated by filtration. The separated black liquor was partially recycled to replace fresh pretreatment liquor in subsequent pretreatment cycles, while the solid fraction was subjected to two-stage disc refining with refining gaps of 0.3 mm and 0.15 mm, respectively, followed by screening to obtain wheat straw CTMP pulp. After five recycling cycles, the solid content of the wheat straw CTMP pretreatment black liquor increased from 6.2% to 14.8%, of which 80.31% consisted of carbohydrate- and lignin-derived degradation products, while the remaining 19.69% comprised inorganic components such as potassium and silicon.

Potassium hydroxide (KOH, AR), hydrochloric acid (HCl, 37%), and sulfuric acid (H_2_SO_4_, 98%) were purchased from Nanjing Chemical Reagent Co., Ltd. (Nanjing, China). Sodium bicarbonate (NaHCO_3_, AR), urea (AR), calcium acetate [Ca(CH_3_COO)_2_, AR], and commercial humic acid (CHA, AR) were obtained from Sinopharm Chemical Reagent Co., Ltd. (Shanghai, China). Phenolphthalein (AR) was purchased from Tianjin Chemical Reagent Research Institute Co., Ltd. (Tianjin, China). All reagents were used as received without further purification. All water used was deionized water.

### 2.2. Preparation of Humic-like Acids

**Figure 1:**
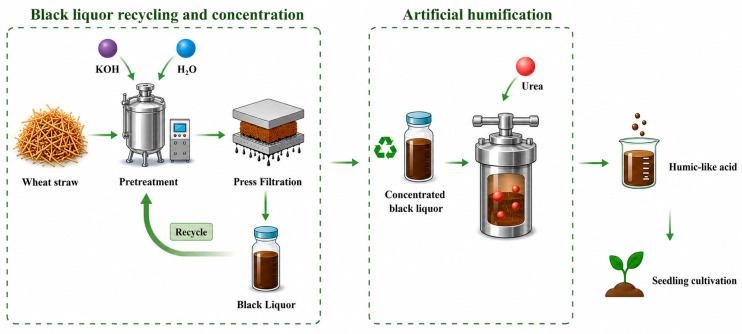
Humification flowchart for recycling wheat straw CTMP black liquor.

During the black liquor recycling process, the pretreated wheat straw slurry was subjected to mechanical press filtration after each pulping cycle to separate the black liquor from the solid fibers. The recovered black liquor (100 mL) was subsequently recycled in the next pulping cycle. After five recycling cycles, a concentrated black liquor was obtained and used as the feedstock for the hydrothermal humification experiments shown in Figure 1.

A total of 50 mL of recycled and concentrated wheat straw CTMP black liquor was transferred into a hydrothermal synthesis reactor (LC-KH-50, Lichen, Shaoxing, China) and subjected to hydrothermal treatment at 140, 160, 180, and 200 °C for 2, 4, 6, and 8 h, respectively. After the reaction, the reactors were cooled to room temperature naturally. The supernatant was acidified to pH 1 using 1.0 mol/L HCl solution and allowed to stand for 2 h to promote precipitation. Subsequently, the mixture was centrifuged using a high-speed centrifuge (H/T16MM, Herexi Instruments, Changsha, China) at 10,000 r/min for 10 min. The obtained precipitate was washed with deionized water and centrifuged repeatedly, followed by drying in a freeze dryer (FDU-2110, Rikakikai, Tokyo, Japan) to obtain a brown powder, which was defined as humic-like acid (HLA). The E_4_/E_6_ ratio, defined as the ratio of absorbance at 465 nm to that at 665 nm (E465/E665) for HLA samples dissolved in 0.05 mol/L NaHCO_3_ solution, is widely used as an indicator of the degree of humification, aromatic condensation, and molecular complexity of humic substances. In this study, the optimal hydrothermal humification conditions, including reaction temperature and reaction time, were comprehensively determined based on HLA yield, total acidic functional group content, and the E_4_/E_6_ ratio, which was measured using a dual-beam UV–visible spectrophotometer (TU-1900, Purkinje General, Beijing, China).

Under the optimal hydrothermal conditions, urea was introduced into the recycled KOH-based wheat straw CTMP black liquor as an external nitrogen source, and the urea concentrations were adjusted to 0.5, 1.0, and 1.5 mol/L, respectively. Hydrothermal reactions were then conducted again under the optimized temperature and time conditions to prepare nitrogen-enriched humic-like acids (N-HLA). According to the urea concentration in the reaction system, the obtained products were designated as 0.5N-HLA, 1N-HLA, and 1.5N-HLA, respectively. The yield of humic-like acids was calculated according to Equation (1).

(1) $$Y_{HLA}=\frac{W_{HLA}}{W_{BL}}\times 100\%$$

Y_HLA_ and W_HLA_ represent the yield of HLA (%) and the mass of HLA obtained after acidification precipitation (g), respectively. W_BL_ represents the mass of total solids in the black liquor (g).

### 2.3. Seed Germination Assay

Radish seeds of full and uniform size and without visible disease spots were selected as experimental materials. The seeds were repeatedly washed five times with deionized water to remove surface impurities and then air dried at room temperature to constant weight for subsequent use. For each treatment, 80 seeds were evenly placed in a Petri dish with dimensions of 12 cm × 12 cm × 6 cm. Germination was conducted in Petri dishes lined with three layers of sterile filter paper at the bottom to prevent moisture loss. The prepared HLA solid samples were dissolved in 0.1 mol/L KOH solution. After complete dissolution, the solution pH was adjusted to 7 using 0.1 mol/L HCl solution, and the HLA solution was subsequently diluted with deionized water to different concentrations of 10, 25, 50, 100, 200, 300, and 400 mg/L. Deionized water treatment was used as the control group (CK). The seeds were soaked in HLA solutions of different concentrations and incubated in the dark at 26 °C for 48 h. Seed germination was defined as the radicle breaking through the seed coat and elongating to 1–2 mm. After the incubation period, the germination rate of each treatment group was recorded, and the root length of seedlings was measured to evaluate the effects of different HLA concentrations on seed germination. The relative germination index (GI) of seeds was calculated according to the following equation:

(2) $$GI=\frac{{GR}_{HLA}\times {RL}_{HLA}}{{GR}_{CK}\times {RL}_{CK}}\times 100\%$$

GR_CK_ represents the seed germination rate in the control group (%). RL_CK_ represents the root length in the control group (cm). GR_HLA_ represents the seed germination rate in the treatment group (%). RL_HLA_ represents the root length in the treatment group (cm).

### 2.4. Seedling Cultivation Experiment

Based on the results of the seed germination experiment, the optimal treatment concentration was determined as the HLA concentration corresponding to the highest germination index. Radish seeds were separately soaked in the selected HLA solution and deionized water (control group) at 26 °C for 48 h. Subsequently, the germinated seeds were uniformly sown into seedling trays and cultured in an electro-thermostatic incubator (YT-100L, Yuntang, Weifang, China). The environmental conditions were set as follows: temperature 26 ± 1 °C, relative humidity 70% ± 10%, and photoperiod of 16 h/day.

During the cultivation period, 15 mL of the corresponding treatment solution (HLA solution or deionized water) was added daily to maintain substrate moisture and ensure treatment consistency. After 14 days of cultivation, seedlings with uniform growth status were selected for sampling. Relevant growth parameters and physiological–biochemical indicators were measured to evaluate the growth-promoting effects of humic-like acids on plants.

### 2.5. Characterization

The total acidic functional group content in humic-like acids was determined by titration. Briefly, 0.0450 g of the HLA sample was added to 2 mL of 0.1 mol/L calcium acetate solution, and the mixture was rapidly sealed under nitrogen protection. After standing and reacting at room temperature for 48 h, 2 mL of 0.1 mol/L HCl solution was quickly added to the centrifuge tube to terminate the reaction, followed immediately by filtration. Two drops of phenolphthalein indicator were then added to the filtrate, and the solution was subsequently titrated with a standard 0.1 mol/L NaOH solution. The consumption volume of the NaOH standard solution during titration was recorded and calculated according to Equation (3). In addition, the lignin content in acid-soluble fractions of black liquor was also determined. The changes in organic components in the enriched black liquor and in black liquor before and after high-temperature humification were analyzed by high-performance liquid chromatography (HPLC, Agilent 1200, Agilent Technologies, Santa Clara, CA, USA). Elemental composition (C, H, O, N) of humic-like acid samples was determined using an elemental analyzer (2400 II, Perkin Elmer, Waltham, MA, USA), and the oxygen content was calculated by the difference. Functional group characteristics of humic-like acid samples were analyzed by Fourier transform infrared spectroscopy (FT-IR, VERTEX 80 V, Bruker, Karlsruhe, Germany) and X-ray photoelectron spectroscopy (XPS, Axis Ultra DLD, Shimadzu, Kyoto, Japan). XPS measurements were conducted using a monochromatic Al Kα X-ray source (1486.6 eV) operated at 12 kV and 8 mA, corresponding to a power of 96 W. The analyzed area was approximately 700 μm × 300 μm. Binding energy calibration and charge correction were performed using the C–C/C–H peak at 284.8 eV. The microstructure of humic-like acid samples was examined using field emission scanning electron microscopy (FE-SEM, Regulus 8100, Hitachi, Tokyo, Japan). Particle size distribution was measured using a laser particle size analyzer (Zetasizer Nano, Malvern Panalytical, Malvern, UK), and each sample was tested in triplicate.

(3) $$Total\;acidic\;functional\;groups\;(mmol/g)=\frac{(V_{2}-V_{1})\times c}{m}$$

c represents the concentration of NaOH used for titration (mmol/mL). V_2_ represents the volume of NaOH consumed during titration of the sample (mL). V_1_ represents the volume of NaOH consumed during titration of the blank sample (mL). m represents the mass of the sample (g).

Generative artificial intelligence (ChatGPT, https://chatgpt.com, accessed on 15 May 2026, OpenAI, San Francisco, CA, USA) was used to support the drawing of Figure 1 in this paper, providing only layout suggestions and visual optimization.

## 3. Results and Discussion

### 3.1. Effects of Hydrothermal Temperature and Reaction Time on the Yield of HLA, Total Acidic Functional Groups, and the Degree of Humification

**Figure 2:**
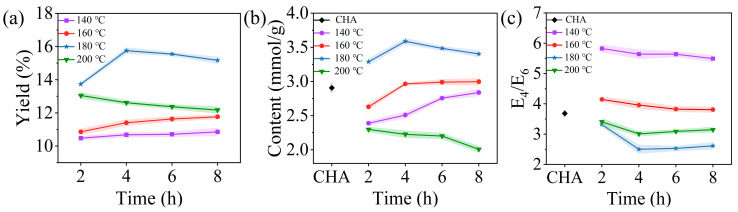
Effects of reaction temperature and time on HLA. (**a**) HLA yield, (**b**) total acidic functional group content, and (**c**) E_4_/E_6_ ratio.

The yields of HLA, total acidic functional groups, and E_4_/E_6_ ratios under different hydrothermal temperatures and reaction times are shown in Figure 2. The yield of HLA is presented in Figure 2a. At 140 °C, the HLA yield remained relatively low, increasing only slightly from 10.48% to 10.85% with prolonged reaction time. When the temperature increased to 160 °C, the HLA yield improved, rising from 10.86% to 11.76% as the reaction time was extended. At 180 °C, the HLA yield further increased, reaching a maximum value of 15.75% at 180 °C for 4 h. This value is higher than that reported by Yang et al. [21], who obtained HLA yields of 1.2% and 1.8% from two types of biomass wastes (wood powder and eucalyptus powder) at 200 °C for 24 h, demonstrating the advantage of using recycled and concentrated black liquor as the feedstock for hydrothermal humification in this study. This temperature condition facilitates the synergistic progression of depolymerization and repolymerization reactions, consistent with previous reports on lignin thermal conversion and humification processes [22]. However, when the temperature further increased to 200 °C or the reaction time was extended to 6–8 h, the HLA yield decreased, reaching 12.18% at 200 °C for 8 h. Under high temperature and prolonged reaction conditions, more intensive dehydration and decarboxylation reactions occur, and excessive condensation may lead to the formation of high-molecular-weight char-like by-products, thereby reducing the recoverable solid yield. Therefore, from the perspective of yield, 180 °C for 4 h is considered the optimal humification condition for this system.

The total acidic functional group content of HLA is shown in Figure 2b. Compared with commercial humic acid (CHA), which exhibits a total acidic functional group content of 2.91 mmol/g, most HLA samples prepared under appropriate hydrothermal conditions show comparable or higher values, with some reaching up to 3.59 mmol/g. This indicates that recycled black liquor under alkaline hydrothermal conditions possesses strong condensation reactivity and a high potential for introducing oxygen-containing functional groups, enabling the formation of humic-like substances with high structural activity. At 140 °C, the total acidic functional group content remained at a relatively low level overall. When the temperature increased to 160 °C, the total acidic functional group content increased significantly, reaching 2.99 mmol/g at 160 °C for 6 h and then tending to stabilize with increasing reaction time. At 180 °C, the total acidic functional group content reached its maximum value, particularly achieving 3.59 mmol/g at 180 °C for 4 h, which is higher than that of CHA. Under sealed alkaline conditions (pH = 9.5) and 180 °C hydrothermal treatment, the humification of organic components in black liquor mainly follows a “condensation–aromatization” pathway [23]. However, when the reaction time is further extended to 6–8 h or the temperature is increased to 200 °C, the total acidic functional group content shows a decreasing trend (2.01–2.29 mmol/g). This indicates that the system gradually shifts from humification-dominated processes to carbonization-dominated pathways. Under higher temperature and longer reaction conditions, decarboxylation reactions are significantly enhanced [24], leading to a reduction in carboxyl groups. Meanwhile, further aromatization and condensation occur, forming denser structures with lower oxygen content and char-like or carbonized products. In addition, part of the phenolic hydroxyl groups participates in condensation reactions to form ether bonds or C–C bonds, resulting in a decreased number of titratable acidic sites.

As shown in Figure 2c, the E_4_/E_6_ ratio of CHA is 3.68, which falls within the typical range of soil humic acids (3–5), indicating a relatively high degree of aromatization, larger molecular weight, and a stable molecular structure. In contrast, the E_4_/E_6_ values of the HLA samples prepared in this study exhibit a clear dependence on reaction conditions. At 140 °C, all HLA samples show higher E_4_/E_6_ ratios than CHA, which decrease with increasing reaction time but remain above 5.45, indicating a relatively low degree of humification. At 160 °C, the E_4_/E_6_ ratios decrease and gradually approach those of CHA. At 180 °C, the E_4_/E_6_ values of all samples further decrease, reaching 2.50 at 4 h, which is lower than that of CHA, indicating that the HLA exhibits a higher degree of aromatization and humification comparable to or even exceeding that of CHA. At 200 °C, the E_4_/E_6_ ratios after 2 h became higher than those observed in the optimal 180 °C range. Overall, the HLA obtained under the optimal condition of 180 °C for 4 h exhibits a lower E_4_/E_6_ ratio and a higher degree of humification.

Based on a comprehensive evaluation of HLA yield, total acidic functional group content, and E_4_/E_6_ ratio, the optimal hydrothermal humification conditions for wheat straw CTMP recycled and concentrated black liquor were determined to be 180 °C and 4 h.

### 3.2. Component Analysis of Hydrothermal Humification of Wheat Straw CTMP Black Liquor

The acidified supernatants obtained after hydrothermal treatment of black liquor systems with different urea concentrations followed by solid–liquid separation were subjected to HPLC compositional analysis. The results are presented in Table 1. As shown in Table 1, the contents of various low-molecular-weight organic compounds in the supernatant of black liquor changed significantly after the high-temperature humification reaction. Overall, the contents of sugars and certain oxygen-containing organic compounds in the post-reaction supernatant decreased markedly, indicating that under the condition of 180 °C for 4 h, the soluble organic fractions in the black liquor were substantially consumed and the system had entered a relatively advanced humification stage.

In the recycled and concentrated wheat straw CTMP black liquor, the contents of xylose and arabinose were 21.05 g/L and 8.08 g/L, respectively, together with a certain amount of glucose. After the hydrothermal reaction, the contents of all monosaccharides decreased significantly, with xylose reduced to 2.28 g/L and arabinose reduced to 0.21 g/L, while glucose was below the detection limit of HPLC after the reaction. These sugars can be converted into key intermediates such as furfural and 5-hydroxymethylfurfural via dehydration reactions. The original 5-hydroxymethylfurfural and furfural present in the black liquor were significantly reduced or not detected after the reaction, indicating that these intermediates can further undergo condensation and polymerization under alkaline hydrothermal conditions, gradually forming humic substances enriched in aromatic structures [25]. In nitrogen-enriched systems, sugars can also react with ammonia or amino-containing species generated from urea decomposition through Maillard-like condensation reactions, thereby promoting nitrogen incorporation and accelerating the formation of humic-like acids [26].

The acetic acid content in the raw black liquor was 11.81 g/L, which decreased to 7.35 g/L after the reaction, and remained relatively stable in the range of 6.04–6.77 g/L under different urea concentrations, showing only minor variation. In contrast, formic acid decreased from 1.94 g/L to 1.62 g/L, indicating that it is more prone to further transformation under alkaline hydrothermal conditions. In addition, the content of levulinic acid and other intermediates was generally low and showed a decreasing trend with urea addition, suggesting their further participation in subsequent transformation processes. Meanwhile, the content of certain alcohols (e.g., glycerol) also decreased, indicating that they may also participate in dehydration or oxidation reactions and be converted into more reactive intermediates.

Notably, although these low-molecular-weight compounds were largely consumed during the reaction, the yield of humic-like acids (HLA/N-HLA) in this study was only about 16%, indicating that not all degradation products were converted into target humic substances. According to the findings of Wang et al. [27], during hydrothermal humification, small molecular intermediates may follow multiple competing reaction pathways in addition to contributing to humic substance formation, including conversion into fulvic acid, further degradation into CO_2_ and H_2_O, rearrangement into low-molecular-weight organic acids, or excessive polycondensation to form water-soluble colloids and highly aromatic carbonaceous materials that are difficult to precipitate under acidic conditions. These side reactions collectively reduce the overall yield of recoverable humic-like acids.

### 3.3. Effects of Urea Addition on the Yield, Total Acidic Functional Groups, and Humification Degree of HLA in Wheat Straw CTMP Black Liquor

**Figure 3:**
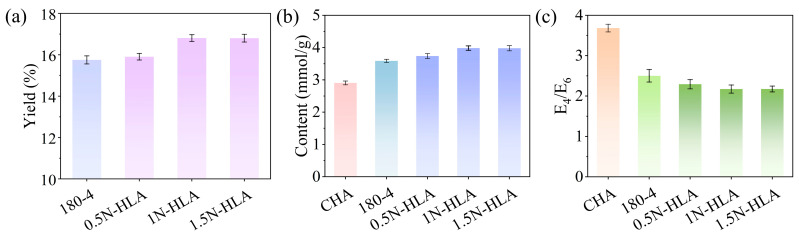
Effect of urea concentration on N-HLA (**a**) yield, (**b**) total acidic functional group content, and (**c**) E_4_/E_6_ ratio.

As shown in Figure 3a, when the urea concentration was 0.5 mol/L, the yield of N-HLA reached 15.90%, which was higher than that of HLA without urea addition. When the urea concentration was increased to 1.0 mol/L, the yield of N-HLA further increased to 16.81%, representing a 1.06% increase compared with the urea-free system, indicating a positive promoting effect of urea on humic-like acid formation. Consistent with Wang et al. [28], nitrogen supplementation can increase the content of nitrogen-containing functional groups and aromaticity of nitrogen-rich artificial humic acids (AHAs) derived from corn straw, while elevating the product yield by 1.99%. Nevertheless, owing to the differences in experimental protocols and raw materials, the yield increment obtained in this work was lower than that reported by Wang et al. However, when the urea concentration was further increased to 1.5 mol/L, the yield remained approximately 16.80%, showing no further increasing trend. The introduction of urea provides an additional ammonia source for the reaction system, enabling part of the low-molecular-weight intermediates to be incorporated into the macromolecular humic structure through crosslinking reactions [29]. In addition, due to chemical interactions between the carbonyl groups of urea and the hydroxyl groups of lignin, native lignin can be dissolved in urea solution. Under the alkaline (KOH) conditions of black liquor, the rigid structure of lignocellulose is disrupted, facilitating urea accessibility and accelerating the degradation of lignin and hemicellulose [26], thereby contributing to the increased yield of N-HLA.

As shown in Figure 3b, the total acidic functional group content of CHA was 2.91 mmol/g, while that of the 180-4 sample was 3.59 mmol/g, which was higher than that of CHA. After the addition of urea, the total acidic functional group content of N-HLA samples further increased. When the urea concentration was 0.5 mol/L, the total acidic functional group content increased to 3.74 mmol/g; when the concentration reached 1.0 mol/L, it reached a maximum value of 3.98 mmol/g; with further increase in urea to 1.5 mol/L, the acidic functional group content remained essentially stable (3.98 mmol/g), with no significant change. Overall, with increasing urea dosage, the total acidic functional group content of N-HLA exhibited an initial increase followed by a plateau, indicating that an appropriate amount of urea promotes the formation of oxygen-containing functional groups during humification, whereas excessive addition has a limited further effect on increasing acidic functionalities.

The E_4_/E_6_ ratios of different N-HLA samples were measured and compared with those of 180-4 and CHA to investigate the effect of urea concentration on structural characteristics, as shown in Figure 3c. The E_4_/E_6_ value of CHA was the highest at 3.68. After hydrothermal treatment of black liquor at 180 °C for 4 h, the E_4_/E_6_ value of the resulting 180-4 sample decreased to 2.50. On this basis, the introduction of urea at different concentrations further reduced the E_4_/E_6_ values of N-HLA, all of which were lower than that of the urea-free system (180-4). This indicates that urea addition further enhances the aromatization and condensation degree of humic-like acid structures. When the urea concentration increased from 0.5 mol/L to 1.0 mol/L, the E_4_/E_6_ value further decreased, suggesting that an appropriate amount of urea facilitates humification reactions and molecular structural complexity. However, when the urea concentration was further increased to 1.5 mol/L, no significant change in E_4_/E_6_ was observed, indicating that the formation and condensation of aromatic structures in the system had reached a relatively stable state.

Based on the comprehensive evaluation of yield, total acidic functional group content, and E_4_/E_6_ ratio, a urea concentration of 1.0 mol/L was identified as optimal for obtaining N-HLA with the most favorable molecular weight characteristics and bioactivity.

### 3.4. Elemental Analysis of HLA

**Figure 4:**
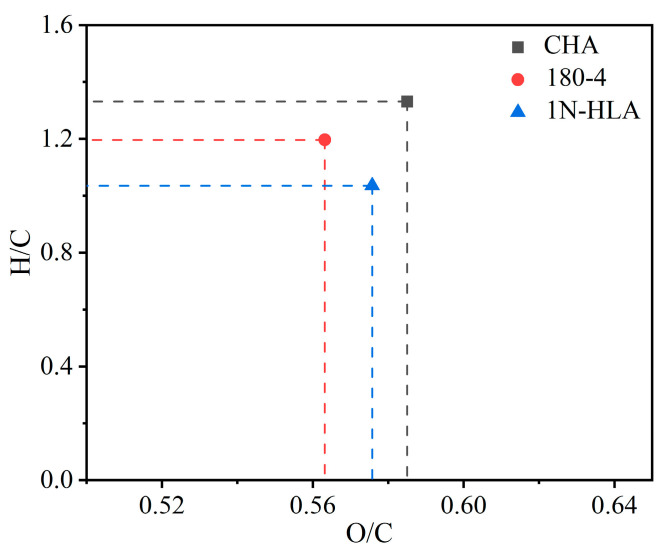
Van Krevelen diagram of CHA, HLA, and N-HLA.

Van Krevelen diagrams, with at.O/C and at.H/C ratios plotted on the *x*-axis and *y*-axis, respectively, are a common tool for characterizing the aromaticity, oxidation degree and structural evolution of humic acids. A decrease in the at.H/C ratio generally reflects enhanced aromaticity and condensation, while a lower at.O/C ratio indicates reduced oxygen-containing functional groups and a decreased oxidation level. The elemental analysis data of CHA, 180-4 and 1N-HLA were further converted into Van Krevelen diagrams, as illustrated in Figure 4.

As can be seen from Figure 4, compared with CHA, 180-4 shifts toward the lower-left region overall, indicating decreases in both at.H/C and at.O/C ratios. In contrast, compared with HLA, the at.H/C ratio of 1N-HLA further decreases after the addition of urea, suggesting an increased degree of condensation and a higher proportion of aromatic structures in the system with the involvement of urea, which is consistent with the trend observed in the E_4_/E_6_ analysis. The at.O/C ratio of 1N-HLA increases to 0.58, indicating that 1N-HLA contains more oxygen-containing functional groups compared with the non-urea system. However, the at.O/C ratios of both HLA samples are still lower than that of CHA. Therefore, the incorporation of an oxidizing agent during the hydrothermal pretreatment process may be considered in future work to further enhance the oxygen content of HLA.

### 3.5. SEM Analysis of HLA

**Figure 5:**
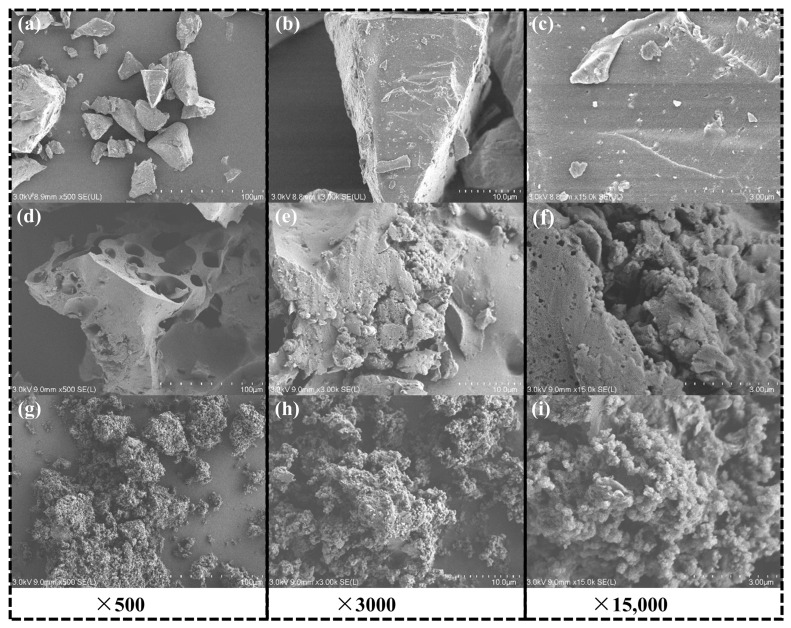
SEM images of CHA (**a**–**c**), 180-4 (**d**–**f**), and 1N-HLA (**g**–**i**) at different magnifications (×500, ×3000, and ×15,000).

As shown in Figure 5, significant changes in the microstructure of different samples can be clearly observed from the SEM images during the hydrothermal reaction and nitrogen-containing humification process. CHA mainly exhibits relatively regular flake-like or block-like structures at different magnifications. The particle edges are relatively smooth, and the overall surface is compact, with only a small number of fine particles locally attached. At higher magnification, the surface still maintains a relatively smooth layered structure with certain lamellar stacking characteristics.

After hydrothermal treatment at 180 °C for 4 h, the microstructure of the obtained HLA is notably different from that of CHA. At ×500 magnification (Figure 5d), a large number of pores and irregular structures can be observed on the surface of the 180-4 sample, exhibiting an overall honeycomb-like porous morphology. At higher magnifications (×3000 and ×15,000, Figure 5e,f), the surface becomes rougher and covered with fine aggregates. These structures are consistent with the self-assembly and condensation of heterogeneous humic-like macromolecules formed during hydrothermal reactions, rather than single-component low-molecular compounds. Based on the combined FT-IR and XPS results, these aggregates are inferred to be a complex mixture of condensed aromatic structures and oxygen-containing functional groups, rather than a single chemical species such as acids or aldehydes. Further compositional identification via other microanalytical techniques will be considered in future work.

After the introduction of urea, the microstructure of the resulting 1N-HLA undergoes further evolution. At ×500 magnification, the overall morphology transforms from the original block-like structure into clearly defined particulate aggregates, accompanied by pronounced agglomeration. At ×3000 and ×15,000 magnifications, it can be observed that these particles are composed of a large number of submicron or nanoscale primary particles, forming a typical porous and aggregated structure.

### 3.6. FT-IR Analysis of HLA

**Figure 6:**
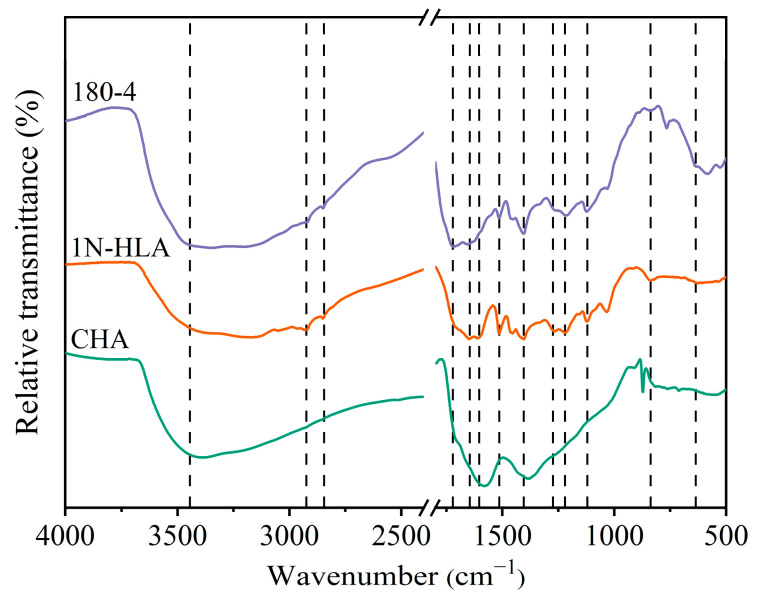
FT-IR spectra of CHA, 180-4, and 1N-HLA.

FT-IR was performed on CHA, 180-4, and 1N-HLA, and the results are shown in Figure 6.

The broad absorption band around 3400 cm^−1^ is mainly attributed to O–H and N–H stretching vibrations. In comparison, the absorption peak in this region becomes broader in 1N-HLA.The stretching vibration adsorption bands of aliphatic C-H bonds are in the 3000–2700 cm^−1^ range [30].

The absorption bands at approximately 2920 cm^−1^ and 2850 cm^−1^ are mainly assigned to the stretching vibrations of aliphatic C–H groups. The relatively weaker intensity of these peaks in 180-4 indicates that part of the aliphatic structures was cleaved or involved in condensation reactions during the hydrothermal process, leading to a gradual enrichment of aromatic structures in the system. In contrast, the stronger absorption in this region observed for 1N-HLA suggests that aliphatic structures are partially retained during nitrogen-containing humification.

The band at 1710–1740 cm^−1^ is ascribed to C=O stretching of carboxyl groups and other carbonyl groups, indicating the presence of carboxyl, ketone, and amide functionalities generated during hydrothermal humification.

The absorption band around 1630 cm^−1^ is mainly attributed to aromatic C=C skeletal vibrations and conjugated carbonyl structures, indicating the formation of aromatic and condensed humic-like macromolecules during hydrothermal humification. Compared with 180-4, 1N-HLA exhibits a more pronounced absorption in the 1630–1660 cm^−1^ region, which can be attributed to amide I (C=O stretching) vibrations formed through the incorporation of nitrogen-containing structures during urea-assisted hydrothermal treatment. The above results reveal that the enrichment patterns of nitrogen-containing functional groups and aromatic structures in the samples are consistent with the FTIR characteristic peaks of humic acids extracted from sediments of various rivers and lakes across China, as reported by He et al. [31].

The absorption region between 1490 and 1590 cm^−1^ contains overlapping contributions from aromatic C=C skeletal vibrations, aliphatic C–H bending vibrations, and nitrogen-containing functional groups [32].

The peak at about 1330 cm^−1^ stands for C-H deformation of CH_2_ and CH_3_ groups, and/or antisymmetric stretching of COO^−^ groups [30]. The persistence of this peak in 180-4 and 1N-HLA indicates that carboxyl-containing structures were retained during hydrothermal humification, contributing to the abundance of acidic functional groups in the humic-like products.

The adsorption intensity of the band at 1228 cm^−1^ is attributed to the C=O stretching of aryl esters and C-O stretching of aryl ethers and phenols [33]. This result suggests that lignin-derived aromatic ether and phenolic structures were incorporated into the humic-like macromolecular framework during hydrothermal humification, thereby enhancing the aromaticity and structural complexity of the products.

The absorption feature in the 1100–1010 cm^−1^ region is attributed to the overlapping contributions of C–O stretching vibrations and, potentially, C–N stretching vibrations from nitrogen-containing functional groups [32]. This band remains evident in both 180-4 and 1N-HLA, indicating the retention of oxygen- and nitrogen-containing structures within the humified products. Combined with the enhanced aromaticity and condensation degree observed in other spectral regions, these results suggest that the HLA samples possess more complex macromolecular structures and a lower proportion of easily degradable components than CHA.

In the range of 900–650 cm^−1^, the humic-like acid samples exhibit a certain absorbance, which is attributed to C–H bending vibrations of substituted aromatic groups, consistent with the spectral features reported for sedimentary humic acids from the Pearl River [31].

### 3.7. XPS Analysis of HLA

**Figure 7:**
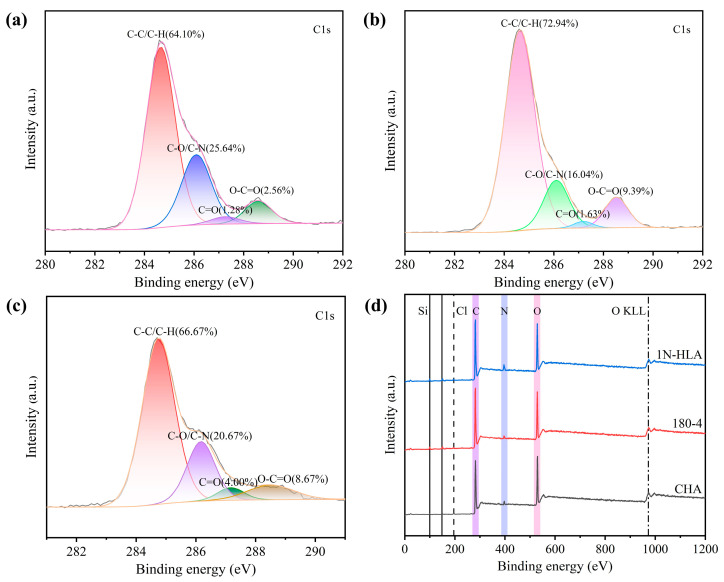
Analysis of C1s spectra of CHA (**a**), 180-4 (**b**), 1N-HLA (**c**), and survey spectrum (**d**).

XPS was employed to analyze CHA, 180-4, and 1N-HLA samples. Figure 7 shows the high-resolution scan spectra of C1s and the survey spectrum of the samples. The C1s signal was deconvoluted into four peaks corresponding to distinct carbon chemical states. The fitted peaks are located at approximately 284.8 eV, 286.1 eV, 287.1 eV and 288.9 eV, which are assigned to aliphatic/aromatic C–C/C–H, C–O/C–N, C=O and O=C–O groups, respectively.

For spectral processing, the Shirley background was used for background subtraction. All peak deconvolutions were performed using pure Gaussian lineshapes, which is suitable for characterizing the electron energy distribution of disordered organic macromolecules. During the fitting procedure, the binding energy of each sub-peak was constrained to the typical values for humic substances and biomass-derived organic compounds as reported in reference [27], ensuring the chemical rationality of peak assignment. No fixed ratio constraints were imposed on the relative intensity (peak area) of different carbon species. Meanwhile, the full width at half maximum (FWHM) of each peak was allowed to vary freely within a reasonable range of 0.9–1.2 [34], so as to minimize the deviation between the fitted curve and experimental data.

As shown in Figure 7a, carbon in the CHA sample is primarily present in the form of C–C/C–H, accounting for 64.10%, indicating that CHA contains abundant aliphatic and/or aromatic carbon skeleton structures. The relative content of C–O/C–N is 25.64%, which can be mainly attributed to alcoholic hydroxyl groups, ether bonds, or carbon atoms bonded to nitrogen. The O–C=O component accounts for 2.56%, corresponding to carbon atoms in carboxyl or ester groups, which are typical oxygen-containing functional groups in humic-like acid materials. From the C1s spectrum of 180-4, the relative content of C–C/C–H is higher than that of CHA, while the C–O/C–N content is 16.04% and the O–C=O content increases to 9.39%, indicating that 180-4 contains more abundant acidic functional groups. After the introduction of urea in the HLA preparation process, the resulting 1N-HLA sample shows further structural evolution. From the survey spectrum (d), the intensity of the N1s peak in 1N-HLA is higher than that in both CHA and 180-4, confirming the successful incorporation of nitrogen derived from urea. Moreover, the relative content of C–N in 1N-HLA is further increased compared with 180-4. In terms of yield, the addition of 1.0 mol/L urea increased the 1N-HLA yield to 16.81%.

The XPS survey spectrum reveals that C, O and N are the predominant elements in all three samples, accompanied by minor silicon signals derived from silicate species and inorganic ash impurities in the raw black liquor. As illustrated in Figure 7d, weak Si 2p, Si 2s and Cl 2p peaks are additionally identified. Specifically, silicon species stem from inherent silica and silicates in wheat straw feedstock, whereas chloride originates from residual chloride ions during the acid precipitation procedure for humic acid purification. A typical O KLL Auger peak, a common feature in XPS analysis, is also present at 900–1100 eV. Given the low intensities of these impurity peaks, such inorganic components only exist at trace levels and exert negligible effects on the structural characterization of humic-like acids.

The XPS results provide molecular-level evidence for this promoting effect, demonstrating that nitrogen species derived from urea can be incorporated into the humic-like acid framework, leading to the formation of more structurally stable nitrogen-containing humic macromolecules. It is noticeable that the peaks assigned to oxygen-containing carbon groups (C=O and O–C=O) exhibit larger full widths at half maximum (FWHM) than those of C–C/C–H groups across all XPS spectra. This is a typical characteristic of humic-like substances, which originates from the diverse chemical microenvironments of various oxygen-containing functional groups within the molecular framework [35]. The coexistence of carboxylic acids, esters, lactones and conjugated carbonyls leads to signal overlap and peak broadening, owing to their slightly varied binding energies [36]. For the 1N-HLA sample specifically, the O–C=O peak presents an even broader profile. Urea doping introduces abundant nitrogen-containing functional groups including amides and amino groups, which form extensive intermolecular hydrogen bonds with adjacent O=C–O structures. Combined with the increased molecular condensation degree after hydrothermal treatment, the chemical environments of carboxyl and ester moieties become more complex, further widening the O–C=O peak [37]. In addition, the low relative content of C=O and O–C=O groups, along with the limited spectral resolution of the instrument, also contribute to the peak broadening phenomenon.

### 3.8. Particle Size Analysis of HLA

**Figure 8:**
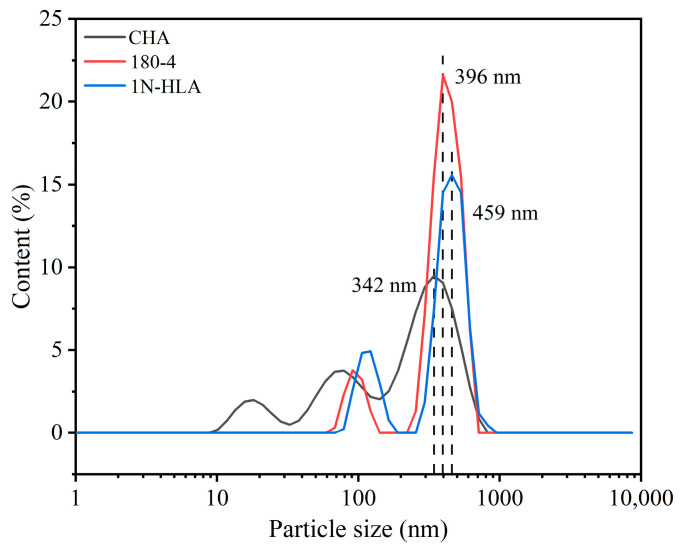
Effect of urea addition on particle size of artificial humic acid. The vertical dotted lines mark the peak particle sizes corresponding to the main peaks of the particle size distribution curves.

Particle size is one of the key physical parameters that significantly influence the interfacial properties, reactivity, and application performance of humic-like acid substances. To investigate the effects of hydrothermal treatment and urea modification on the particle size distribution of humic-like acid products, a laser particle size analyzer was employed to determine the particle size distributions of CHA, 180-4, and 1N-HLA, and the results are shown in Figure 8. The particle size distribution curves indicate obvious differences among the samples. The CHA sample exhibits a relatively broad particle size distribution, with its main peak centered at approximately 342 nm, along with a weak peak in the smaller particle size range. After hydrothermal treatment at 180 °C, the main peak of the 180-4 sample shifts to 396 nm. With further addition of urea, the main peak of the particle size distribution of the obtained 1N-HLA sample continues to shift toward larger particle sizes, with an average particle size of 459 nm. Meanwhile, the introduction of nitrogen-containing functional groups enhances intermolecular hydrogen bonding and crosslinking interactions, facilitating the formation of larger aggregated structures of humic-like molecules. As a result, an increase in particle size is observed in the particle size distribution [38].

### 3.9. Effects of HLA on Seed Germination

**Figure 9:**
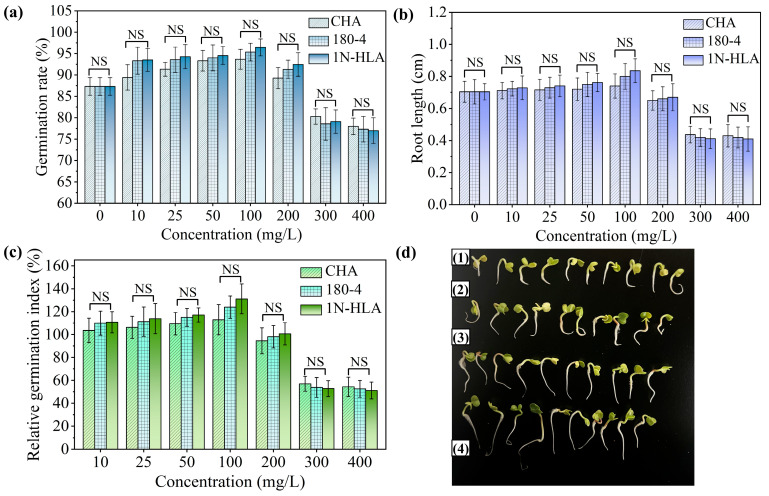
The effect of humic-like acids on promoting seed germination. (**a**) Seed germination rate; (**b**) radicle length; (**c**) germination index; (**d**) seed germination morphology. (**1**), (**2**), (**3**), (**4**) represent treatments with deionized water, CHA, 180-4, and 1N-HLA, respectively. NS indicates no significant difference between treatments at the same concentration (one-way ANOVA followed by Tukey’s HSD test, *p* > 0.05).

Figure 9 illustrates the effects of different concentrations of humic-like acids (CHA, 180-4 and 1N-HLA) on the germination rate, root length and relative germination index of radish seeds, with deionized water serving as the blank control.

As shown in Figure 9a, all humic-like acid treatments exerted a positive effect on radish seed germination, and the promoting efficiency presented an obvious dose-dependent relationship. For all experimental groups, the seed germination rate increased gradually as the concentration rose from 0 to 100 mg/L and reached the maximum value at approximately 100 mg/L, suggesting that humic-like acids facilitate seed germination at relatively low concentrations. When the concentration was further increased to 200–400 mg/L, the germination rate began to decline, indicating that excessively high concentrations of humic-like acids would inhibit seed germination. This trend is consistent with the well-documented pattern of low-concentration stimulation and high-concentration inhibition for artificial humic substances reported by Yang et al. [39] in plant physiological studies. Under low-concentration conditions, the abundant carboxyl, phenolic hydroxyl, and quinone functional groups in humic acid molecules can facilitate water uptake by seeds and enhance cell membrane permeability. Meanwhile, they can also activate key enzymes involved in seed germination (such as amylase and peroxidase), thereby accelerating the degradation and transport of storage substances in the endosperm and promoting radicle emergence through the seed coat to complete germination [40]. In contrast, under high-concentration conditions, the relatively high proportion of aromatic structures and phenolic compounds in humic acid molecules may increase the osmotic pressure of the culture system and impose physiological stress on radicle cells, thereby leading to a decrease in germination rate.

As shown in Figure 9b, within the low to medium concentration range (25–100 mg/L), all humic acid treatments promoted radicle elongation, with 1N-HLA exhibiting the most pronounced effect, showing higher root length than both CHA and 180-4 treatments. This indicates that nitrogen-enriched humic-like acids not only promote seed germination but also effectively stimulate root cell division and elongation. Previous studies have demonstrated that humic acids can regulate the levels of auxin-like substances in plants and enhance plasma membrane H^+^-ATPase activity, leading to extracellular acidification and cell wall loosening, which facilitates cell elongation and thus promotes root growth and lateral root formation [41]. When the humic acid concentration exceeded 200 mg/L, root length showed a decreasing trend.

As presented in Figure 9c, the variation trend of the relative germination index was generally consistent with that of germination rate and root length, increasing significantly at medium concentrations and decreasing at high concentrations. Among all treatments, 1N-HLA exhibited the highest germination index at approximately 100 mg/L, indicating that nitrogen-doped humic-like acids can enhance seed germination vigor under appropriate concentration conditions.

At identical concentration levels, the structural and functional group differences among the three samples (CHA, 180-4, and 1N-HLA) only induced minor numerical disparities in the regulation of germination rate, root length, and relative germination index, and these gaps failed to reach statistical significance (*p* > 0.05). Nevertheless, descriptive mean values consistently revealed that 1N-HLA yielded slightly superior performance in germination rate, root length, and relative germination index compared with the control commercial humic acid CHA and the urea-free hydrothermally synthesized sample 180-4; this subtle advantage remained stable across the concentration range of 25–200 mg/L. These observations indicate that 1N-HLA derived from pulp and papermaking black liquor exhibits a potential tendency to facilitate radish seed germination and radicle growth at a level comparable to or even better than CHA.

Overall, within the optimal concentration range, the humic-like acids synthesized in this study exhibited prominent growth-promoting effects on the germination rate, root length and relative germination index of radish seeds. Specifically, the relative germination index of radish seeds treated with 1N-HLA reached 131.14%. This result is well consistent with the report by Wang et al. [27] where humic-like acids obtained from composting increased the relative germination index of corn seeds to 132%.

### 3.10. Effect of HLA on Seedling Growth

**Figure 10:**
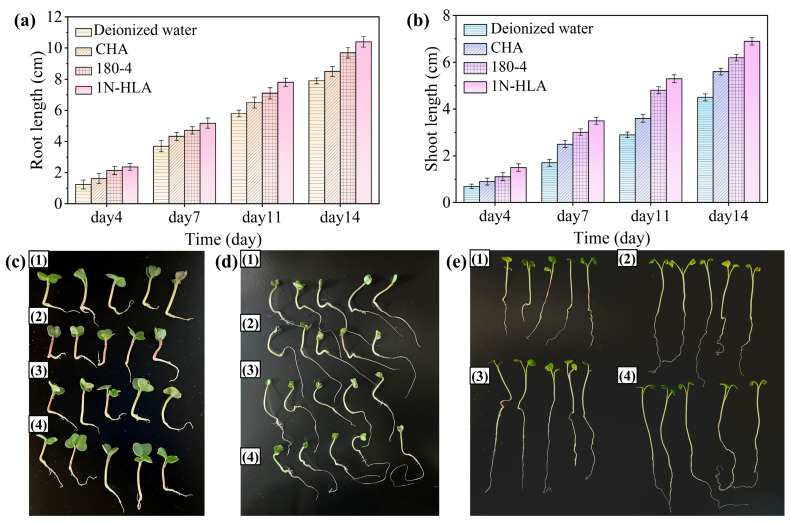
The effect of humic-like acid on promoting seedling growth. (**a**) Root length; (**b**) stem length; (**c**) seedling growth Day 4; (**d**) seedling growth Day 7 and (**e**) seedling growth Day 11. (**1**), (**2**), (**3**), (**4**) represent treatments with deionized water, CHA, 180-4, and 1N-HLA, respectively.

At an appropriate concentration (100 mg/L), the effects of humic-like acids derived from different sources on radish seedling growth were further investigated, as shown in Figure 10. The root length and shoot length of seedlings in all treatment groups showed a continuous increasing trend with prolonged cultivation time, indicating that seedlings developed normally after germination in the experimental system and that the experimental conditions were stable and reliable. Since the root systems were well developed and numerous lateral roots had formed at Day 14, making it difficult to capture complete images of intact plants, only seedlings from Day 4 to Day 11 were photographed.

Comparative analysis among different treatments revealed that the promoting effects of the three humic acid materials on seedling growth followed the order: 1N-HLA > 180-4 > CHA > deionized water. Both root length and shoot length in the 1N-HLA treatment were higher than those in the other groups, demonstrating a stronger growth-promoting effect compared with the 180-4 and CHA treatments. This indicates that the introduction of nitrogen sources during the humification process can effectively enhance the bioactivity of humic-like acids. This phenomenon is related to the fact that abundant carboxyl and phenolic hydroxyl groups in humic acid molecules can promote root cell division and elongation [42]. During hydrothermal humification, the incorporation of urea leads to the formation of nitrogen-containing functional groups, which enhances the nutrient-supplying capacity and molecular reactivity of humic-like acids, activates nitrate reductase and glutamine synthetase, promotes nitrogen uptake and assimilation, and thereby facilitates protein and chlorophyll synthesis, ultimately enhancing photosynthetic efficiency and promoting shoot growth [43].

Under appropriate concentration conditions, humic-like acids can promote the growth of radish seedlings, among which nitrogen-doped humic-like acids (1N-HLA) exhibit the most pronounced growth-promoting effect. This suggests that introducing nitrogen sources during the hydrothermal humification process can effectively improve the structure of humic-like acids and enhance their agricultural performance, providing important theoretical support for the valorization and high-value conversion of pulping and papermaking waste liquors.

## 4. Conclusions and Limitations

### 4.1. Conclusions

This study used concentrated black liquor obtained after five cycles of recycling KOH-pretreated wheat straw CTMP black liquor as the raw material. The enriched organic components and residual alkalinity in the system were fully utilized to convert organic fractions into HLA via hydrothermal reactions. On this basis, urea was introduced for nitrogen modification to obtain nitrogen-enriched humic-like acid (N-HLA). The yield variations and structural characteristics of the products were systematically investigated. The hydrothermal pretreatment conditions and urea concentration were optimized, and the structures of the prepared HLA and N-HLA were further analyzed.

(1) The optimal alkaline hydrothermal humification conditions for wheat straw CTMP black liquor were 180 °C for 4 h, under which the yield of HLA reached 15.75%. Under the same condition (180 °C, 4 h), the optimal urea dosage was 1 mol/L, and the yield of 1N-HLA reached 16.81%.

(2) The total acidic functional group content of HLA obtained from concentrated wheat straw CTMP black liquor treated at 180 °C for 4 h was higher than that of CHA. Under urea addition, the total acidic functional group content of the 1N-HLA sample further increased. The E_4_/E_6_ ratios indicated that low-molecular-weight organic compounds gradually transformed into high-molecular-weight aromatic structures within the system. EA showed that the at.H/C and at.O/C ratios of the humic-like acid samples were lower than those of CHA, indicating higher aromaticity and a higher degree of condensation in the synthesized products. SEM and PSA results demonstrated that humic-like acid molecules continuously polymerized during the reaction to form stable aggregates. FT-IR analysis indicated that the vibrational peaks of the aromatic skeleton were significantly enhanced after hydrothermal treatment, while oxygen-containing functional groups such as carboxyl and hydroxyl groups underwent partial rearrangement and transformation. XPS confirmed that nitrogen-containing active species generated from urea decomposition participated in the formation of nitrogen-containing functional groups and were incorporated into the humic-like acid molecular framework.

(3) At a concentration of approximately 100 mg/L, both humic-like acids (180-4 and 1N-HLA) promoted radish seed germination and root length growth. In terms of seedling growth, all humic-like acid treatments promoted growth to varying degrees, following the order: 1N-HLA > 180-4 > CHA > control group (deionized water).

### 4.2. Limitations and Future Work

Although this study has demonstrated the feasibility of converting concentrated black liquor from recycled KOH-pretreated wheat straw chemi-thermomechanical pulping (CTMP) into humic-like acids (HLA) and nitrogen-enriched humic-like acids (N-HLA) via hydrothermal humification, several limitations and directions for future work remain to be addressed.

The structural evolution of HLA and N-HLA was characterized using elemental analysis, FT-IR, XPS, SEM, and particle size analysis. Collectively, these techniques provided evidence for enhanced aromatization, molecular condensation, and successful nitrogen incorporation during hydrothermal treatment and urea modification. However, more comprehensive characterization methods, particularly energy-dispersive X-ray spectroscopy (EDS), solid-state ^13^C nuclear magnetic resonance (NMR), and ^15^N NMR, could offer direct molecular-level information regarding carbon skeleton transformation, aromatic carbon condensation, and the distribution of nitrogen-containing functional groups. Therefore, future research should employ solid-state NMR and other advanced characterization techniques to further elucidate the humification mechanism and nitrogen incorporation pathways.

Although the hydrothermal conditions and urea dosage were systematically optimized in this study, and CHA was used as a control for comparison with the as-synthesized humic-like acid samples, a control group using concentrated black liquor combined with urea without hydrothermal treatment was not included. This additional control would help differentiate the individual contributions of hydrothermal humification and urea addition alone. Further studies will establish such control systems to comprehensively clarify the roles of hydrothermal reaction and nitrogen modification in the formation of N-HLA.

The biological activity assessment was limited to radish seed germination and early seedling growth. While the results demonstrated the growth-promoting potential of N-HLA, more extensive evaluations involving different crop species, growth stages, soil conditions, and application methods are required to fully assess its agricultural performance and practical applicability.

Future research should integrate techno-economic analysis, life cycle assessment, and cross-comparisons between different feedstocks to evaluate the competitiveness and industrial potential of black liquor-derived humic-like materials.

Overall, this work provides a promising pathway for the high-value utilization of non-wood pulping black liquor. Nevertheless, further mechanistic studies and scale-up evaluations are necessary to support the development of sustainable and industrially viable humic-like products.

## Figures and Tables

**Table 1:** Chemical composition of wheat straw CTMP black liquor and the supernatants after hydrothermal humification.

Ingredient (g/L)	Concentrated Black Liquor	180-4	0.5N-HLA	1N-HLA	1.5N-HLA
Glucose	3.66 ± 0.04	NA	NA	NA	NA
Xylose	21.05 ± 0.17	2.28 ± 0.04	1.64 ± 0.03	1.28 ± 0.05	1.28 ± 0.03
Arabinose	8.08 ± 0.11	0.21 ± 0.02	0.17 ± 0.002	0.07 ± 0.001	0.07 ± 0.005
Cellobiose	2.79 ± 0.08	0.19 ± 0.03	NA	NA	NA
Acid-soluble lignin	15.71 ± 0.14	NA	NA	NA	NA
Acid-insoluble lignin	38.93 ± 0.31	NA	NA	NA	NA
Formic acid	4.06 ± 0.09	1.94 ± 0.06	1.64 ± 0.04	1.62 ± 0.05	1.60 ± 0.04
Acetic acid	11.81 ± 0.17	7.35 ± 0.08	6.77 ± 0.06	6.07 ± 0.07	6.04 ± 0.05
Levulinic acid	0.15 ± 0.03	0.11 ± 0.004	0.08 ± 0.001	0.06 ± 0.001	0.06 ± 0.005
5-Hydroxymethylfurfural	0.26 ± 0.02	0.03 ± 0.01	NA	NA	NA
Furfural	0.33 ± 0.005	0.01 ± 0.005	NA	NA	NA
Furan	0.22 ± 0.03	0.07 ± 0.002	0.04 ± 0.001	0.03 ± 0.004	0.03 ± 0.003
Ethanol	0.22 ± 0.02	0.13 ± 0.004	NA	NA	NA
Glycerol	0.76 ± 0.02	0.51 ± 0.05	0.39 ± 0.01	0.38 ± 0.04	0.38 ± 0.03

Note: “NA” in the results indicates not detected, and the detection limit for the substances is 0.00001 mg/L.

## Data Availability

Due to privacy restrictions, these data are not publicly available. The data presented in this study are available on request from the corresponding author.
